# Therapeutic Targeting of Histone Deacetylation to Prevent Alzheimer’s Disease

**Published:** 2021-04-26

**Authors:** Sophia Chacko, Warren Ladiges

**Affiliations:** 1 Department of Comparative Medicine, School of Medicine, University of Washington, USA

**Keywords:** geroscience, Alzheimer’s disease, histone deacetylation, histone deactylase inhibitors

## Abstract

Efforts to find disease-modifying treatments for Alzheimer’s disease (AD) have been largely unsuccessful. The relative lack of progress and the age-related incidence of AD suggest that modulation of aging per se may be a useful alternative treatment approach. Therapeutics aimed at preventing or reversing aging should be effective in preventing or reversing dementia and the pathology associated with progressive AD. Epigenetic dysregulation of neuronal gene expression occurs with age, propagating deficits in cellular homeostasis. Regulators of epigenetic processes, such as histone deacetylases (HDACs), are well documented and may represent promising therapeutic targets. HDAC activity becomes dysregulated with age and in AD. An intriguing concept is that HDAC inhibition effectively forestalls AD pathology measured more broadly, addressing the notion that rectifying homeostatic gene expression may be the critical step in ameliorating AD pathogenesis at the earliest stage of disease initiation. HDAC inhibitors target several pathways associated with aging and AD neuropathology including loss of synaptic function, mitochondrial dysfunction, increased oxidative stress, and decreased autophagy activity. Since transcriptional levels of numerous genes are shown to decrease with increasing age, a recovery of their transcriptional activity through HDAC inhibition could prevent or delay age-associated declines in neurological function and provide pathways for treating AD.

## Introduction

The prevalence of neurodegenerative diseases is expected to soar with the number of elderly individuals in both developed and developing countries now rising dramatically. Efforts to find disease-modifying treatments have been largely unsuccessful. These efforts have focused mainly on identifying pathogenic mechanisms specific to each disease process. The relative lack of progress with these approaches and the age-related nature of neurodegenerative disease incidence suggests that modulation of aging per se may be a useful alternative approach for delaying the onset or retarding the progression of neurodegenerative conditions [1,2]. This concept is supported by an impressive body of knowledge identifying genetic, dietary and pharmacologic interventions that profoundly retard aging and its pathophysiologic effects in a number of invertebrate and murine model systems [3]. Correlative human data suggest that these model system results are relevant to humans. Since cognitive decline and Alzheimer’s disease (AD) are highly linked with increasing age as a risk factor, therapeutics aimed at preventing or reversing aging should be effective in preventing or reversing dementia and the pathology associated with progressive AD. A number of molecular targets are well known for influencing aging [4], but only a few have been successfully targeted with individual drugs.

AD is a complex heterogeneous pathology that impacts multiple aspects of neuronal physiology. The predominant hypothesis guiding AD therapeutic development for the last two decades has been that amyloid initiates a cascading series of events, including phosphorylation and aggregation of tau protein, that lead to pervasive neuronal degeneration. The repeated observation that rare early-onset familial AD mutations increase pathogenic amyloid through perturbations of beta/gamma-secretase function, promoted the notion that targeting amyloid is a potential therapeutic approach. Unfortunately, failures of a number of clinical trials have called into question amyloid as the primary therapeutic target. This demands careful reconsideration of AD disease etiology and justifies why alternative strategies for therapeutic development should be explored.

### Targeting Histone Deacetylation

Epigenetic dysregulation of neuronal gene expression occurs with age, propagating deficits in cellular homeostasis. Regulators of epigenetic processes, such as histone deacetylases (HDACs), are well documented and may represent promising therapeutic targets. The gradual and escalating dysfunction of essential cellular processes could link all of the cellular AD phenotypes into a complex cascade, in which amyloid is either a late-stage effector or simply a biomarker of the disease process. In support of this view, numerous transcriptional analysis studies have examined genetic dysregulation in AD and found metabolic processes, oxidative stress, protein degradation, synaptic function and transcriptional regulation to be impacted [5,6]. Gene silencing through chromatin remodeling is one possible mechanism as histone deacetylase (HDAC) expression increases with age for a number of HDAC genes [7], and histone acetylation is altered in AD [8]. Concordantly, HDAC inhibitors restore synaptic viability in AD transgenic mice [9], increase expression of amyloid clearing genes IDE and neprilysin [10] and have positive effects upon memory in AD animal models [11,12]. These studies support the connection between HDAC mediated epigenetic regulation and AD neuronal pathology.

HDAC inhibition is a powerful approach to stimulate transcriptional changes within neurons. HDAC activity becomes dysregulated with age and in AD and has been shown to decrease synaptic plasticity in mouse models [13,14]. The exploration of HDAC inhibitors has been studied in the context of cancer treatment, but there has not been a comprehensive examination of the efficacy of these compounds in preventing dementia and protecting human neurons. An intriguing concept is that HDAC inhibition effectively forestalls AD pathology measured more broadly, addressing the notion that rectifying homeostatic gene expression may be the critical step in ameliorating AD pathogenesis at the earliest stage of disease initiation. The exploration of next generation HDAC inhibitors to treat or prevent AD is innovative because there has not been a comprehensive examination of the efficacy of these compounds in protecting human neurons. This supports a linkage between age-related epigenetic regulation and AD neuronal pathology and the plausibility of treating age-dependent chromatin silencing as a viable therapeutic venue for AD neuropathophysiology. Multiple cellular phenotypes of AD, such as aberrant mitochondrial activity, ROS generation and synaptic dysfunction could be linked to altered metabolism and dysregulated gene expression, suggesting that disruption of gene expression homeostasis may be a primary and early point of disease etiology.

Our published observations that phenylbutyrate (PBA), an HDAC inhibitor, attenuated amyloid plaque development in the PS1delta9/APPswe double transgenic mouse line and partially restored cognitive function [12], set the stage to further investigate HDAC inhibition as a viable approach to treat or prevent AD. PBA is clinically attractive because it is an FDA approved drug, and is safe with relatively few side effects. However, it is cleared rapidly from the blood and is poorly absorbed across the blood brain barrier [15] thus requiring very high and likely problematic doses in patients. A drug that has similar positive effects but with greater potency and greater bioavailability would be more clinically relevant. The HDAC gene family is complex and divided into four classes based on the similarity of structure and mechanism. Recent FDA-approved compounds for treating cancer represent modern HDAC inhibitor chemistries and both broad-spectrum HDAC inhibition (vorinostat, belinostat, and panobinostat) and specific (romidepsin) targeting specificity. Panobinostat (PANB) targets HDAC classes similar to PBA [16], is highly potent, has an existing clinical and safety database [17], efficiently crosses the blood brain barrier, and increases histone acetylation in the brain and other tissues [18].

HDAC inhibitors target several pathways associated with aging and AD neuropathology. Synaptic pathology is increasingly of interest in assessing AD pathology and is linked to normal aging as well [19,20]. Gene expression studies examining transcriptional aberration in AD find that synaptic function appears ubiquitously [21–23], and consequently, refined synaptic pathologies would be expected to be present in both aging and AD, and may be the point of earliest intervention. Neurogranin (NG) and neurofilament light chain (NFL) are informative biomarkers. NFL is a structural component of the cytoskeleton and can be used to assess axonal damage [24,25], while NG is localized to the post-synaptic dendritic spine [26,27]. These two factors specifically examine distinct aspects of synaptic function from a presynaptic and post-synaptic perspective such that rescue of impaired synaptic integrity by HDAC inhibitors could be monitored.

Closely linked with synaptic pathology, mitochondrial dysfunction leading to decreased ATP production and increased ROS resulting from impaired electron transport chain function appears prominently in both aging and AD [28–30]. The ability to non-invasively assess both oxidative and glycolytic metabolism provides a comprehensive measure of neuronal metabolism in AD and non-AD cells, and potential metabolic changes that may impair neuronal function, within one of the most metabolically active cell types in the body [31]. The expectation is that HDAC inhibition would improve mitochondrial function and cellular metabolism in neurons stressed by Abeta 42 and pTau.

Autophagy plays a key role in neuronal physiology and pathology. It degrades cell organelles and misfolded proteins by fusing autophagosomes with lysosomes to prevent buildup of wastes within the cell and promote homeostasis and organelle self-renewal. Misfolded proteins can induce autophagy in primary neurons and this induction can be impaired under neurodegenerative disease conditions [32,33]. Expression of Beclin-1, ATG, LC3B and P62 can be used to determine if there is declining function and imminent apoptosis, or disease states associated with aging and AD pathology. Failure of autophagy may result in development of senescence and enhanced apoptosis and neuronal cell death. HDAC inhibition would expect to stabilize autophagy and enhance healthy neuronal function.

## Conclusion

There is now a growing consensus that increased histone acetylation with globally elevated transcription might be beneficial at older ages as it contributes to reversion of age-dependent decline in expression of metabolic, stress response, and reparative genes involved in homeostasis and health span. There is thus a rationale for targeting deacetylation with inhibitors to activate expression of specific genes. Since transcriptional levels of numerous genes are shown to decrease with increasing age, a recovery of their transcriptional activity through HDAC inhibition could prevent or delay age-associated declines in neurological function and enhance intervention of AD.
